# Sustaining Functionality of Not-for-Profit Health Organisations During Pandemics: Lessons and COVID 19 Experience from Makerere University Walter Reed Project

**DOI:** 10.24248/eahrj.v8i3.800

**Published:** 2025-01-30

**Authors:** Jude Thaddeus Ssensamba, Brenda Atwijuka, Stephanie Nakimuli, Brenda Kyohangirwe, Gladys Atim, Ezra Musingye, Chrispus Musabe Bakunda, Jackline Namugabo, Prossy Naluyima, Albert Musinguzi, Stephen Mugamba, Betty Mwesigwa, Hannah Kibuuka

**Affiliations:** a Makerere University Walter Reed Project, Kampala, Uganda.

## Abstract

Not for profit health organisations (NPHOs) complement government health response efforts, hence the need for their continued functionality during pandemic situations. In this article we highlight lessons from Makerere University Walter Reed Project’s (MUWRP) efforts to ensure continuity of its health mandate during the corona virus disease 2019 (COVID-19) outbreak. Our findings provide cues for other developing world NPHOs as they prepare for the next outbreak. When the first case of COVID-19 was reported in Uganda, MUWRP’s leadership identified four strategic pillars of action; minimising the risk of spread of the malady, ensuring continuity of all health activities, early identification and support for casualties, and prevention. An infection prevention and control (IPC) committee was set up to lead response efforts. Innovations per pillar such as adoption of information technology to ensure virtual working and meeting, bringing vaccines to the doorsteps of interested staff, free COVID testing, alternate employee working schedules, introduction of temperature guns, and weekly IPC review meetings were implemented. Routine demographic, testing, positivity, and treatment data was exported to STATA 15.1 for analysis. By the declaration of the end of the pandemic by the WHO, the average positivity rate of COVID-19 among 196 MUWRP staff was 7%, with 95% of all cases being mild, and 94.3% cases managed through home-based care. Only three cases were referred to hospital. Overall, males 30 to 40 years were most affected. Vaccination completion was at 89%, and there were no fatalities reported. Employing the four pillars and related innovations were key to minimising the effects of COVID-19 at MUWRP and are a relevant adaptable tool for other NPHOs in the developing world, as they prepare for the next pandemic.

## BACKGROUND

Low and middle-income countries (LMICs), more so in Africa, continue to struggle with financing their health care systems.^1^ Whereas some countries such as Malawi, Ethiopia, and Swaziland have been able to meet the Abuja declaration’s African Union heads of state pledge to allocate at least 15% of each country’s annual expenditure to health care, many in the sub Saharan region such as Uganda, Cameroon, Angola, Mali, and Eritrea continue to allocate less than adequate funding to health care in their annual budgets.^2,3^ For this, overseas development assistance (ODA) continues to be a key component of their health care investments.^4,5^ Moreover, experiences of corruption, mal-allocation, and misuse of health development assistance by governments^6–8^ have steered development partners towards international, regional, national, or local not for profit health organizations (NPHOs) to execute country specific health activities on their behalf. ^9,10^

NPHOs fall into the category of legally instituted bodies, institutions, establishments, groups, and initiatives that are fully or largely independent of government whose primary objective is to improve the quality of life of the most in need through a humanitarian noncommercial lens.^11^ Their niche is improvement of the public’s health in the developing world,^12,13^ thus contributing to universal health coverage and access.^14^ Entities such as the World Health Organization (WHO), The Global Fund to Fight AIDS, Tuberculosis and Malaria, Doctors without Borders, AMREF Health Africa, Save the children, World Vision International amongst others, have been credited for improving the health of masses^15–17^ and were more accepted and trusted during pandemics such as the 2014 to 2016 West African Ebola Virus Disease.^18^ In Uganda, nonprofit humanitarian health actors have and continue to be key players in the health sector, and their efforts substantially contribute to the Uganda Vision 2040,^19^ the national development plan,^20^ and the Ministry of Health objectives.^2^ It is thence critical that NPHO operations continue even during public and global health emergencies.

The 2019 COVID pandemic took the world by surprise,^21,22^ including NPHOs that had no prior experience of how operate in such situations. This was compounded by the paucity of information and experiences on how such institutions organize internally to continue with service provision and support to government programs, with minimal risk to their staff. On the 21^st^ of March 2020, Uganda recorded the first COVID-19 case,^23^ and on the 25^th^ of March 2020, Uganda went into a total lockdown.^24^ Although NPHOs were allowed to continue operating, there was lack of clear guidance from the government and international organisations,^25,26^ on how entities such as MUWRP would operate within this environment. This posed a high risk of exposure to its staff, more so those engaged in diseases surveillance, lab, research, community engagement, and supporting day to day running of program activities. In this paper, authors employed a continuous learning phenomenological approach to investigate the efforts, innovations, and learnings by Makerere University Walter Reed Project (MUWRP) [the NPHO] to ensure continuity of its health activities during the COVID-19 pandemic. This experience provides cues to other NPHOs in the developing world on how to prepare and function during the next pandemic.

### About Makerere University Walter Reed Project

The Makerere University Walter Reed Project (MUWRP) is one of Uganda’s leading not-for-profit independent health research and implementation organisation. Established in 2002, the organisation currently has four programs that support the Government of Uganda (GoU) health agenda; (1) Research which conducts vaccine trials and other basic science research, supported by a College of American Pathologists accredited laboratory, (2) the U.S. President’s Emergency Plan for AIDS Relief (PEPFAR) program which provides HIV prevention and treatment services across six districts in central Uganda, (3) the Austere environment Consortium for Enhanced Sepsis Outcomes (ACESO) which leverages novel technologies and medical countermeasures in the management of severe infectious illnesses in austere settings, and (4) the Emerging Infectious Diseases Program (EIDP) which conducts surveillance for human respiratory pathogens, antimicrobial resistance, zoonotic diseases, febrile and vector borne pathogens and causes of acute gastroenteritis ^27^. During the COVID-19 pandemic, the organisation had a total of 196 staff spread across 12 operational sites. MUWRP recorded its first COVID-19 case on July 27, 2020.

## METHODS

This was a prospective quantitative observational inquiry. The COVID-19 outbreak being an unplanned event, there was no systematic process to introduce, implement, study, and modify or withdraw (plan, do, study, act cycle) a particular change/innovation, but all changes were collectively implemented, and sustained throughout the whole pandemic period; while data on outcome such as positivity rates, and severity were collected routinely.

MUWRP’s executive management held its first COVID-19 crisis meeting on 21^st^ March 2020. Deliberations hinged on four pillars: (1) Continuity of organisational activities, (2) Minimisation of the risk of acquisition and spread of the disease among staff within and outside the organisation’s premises, (3) Early identification and support systems for positive staff, and (4) Sensitisation and support for prevention, crosscutting leadership and coordination. By the end of the meeting, a twelve member Infection Prevention and Control committee was instituted to spear head short, medium, and long term actions, changes, and innovations in line with the four themes (Table 1).

**Table 1:** Summary of the Terms of Reference for the IPC Committee

Develop and implement a MUWRP COVID-19 Risk Management Policy
Develop and implement department-specific IPC guidelines in line with the MUWRP COVID-19 risk management policy
Meet online weekly to discuss MUWRP occupational health and safety
Follow up and support symptomatic and sick staff members and their contacts
Collect samples from symptomatic staff for testing at UVRI
Inform, educate, train and regularly update staff on pandemic-related matters
Promote and emphasize prevention and vaccination measures
Support evacuation and referral to health facilities of positive staff
Clear cured staff to return to work
Follow up of positive staff and their families to ensure they have good care

### Thematic Actionable Areas Minimisation of the Risk of Acquisition and Spread of the COVID-19

Risk minimisation was the overarching objective for ensuring continuity of all research, surveillance, and implementation science activities. For this, the organisation adopted structural, technological, and human resources changes.

#### Structural Changes/Enhancements

**Figure 1:**
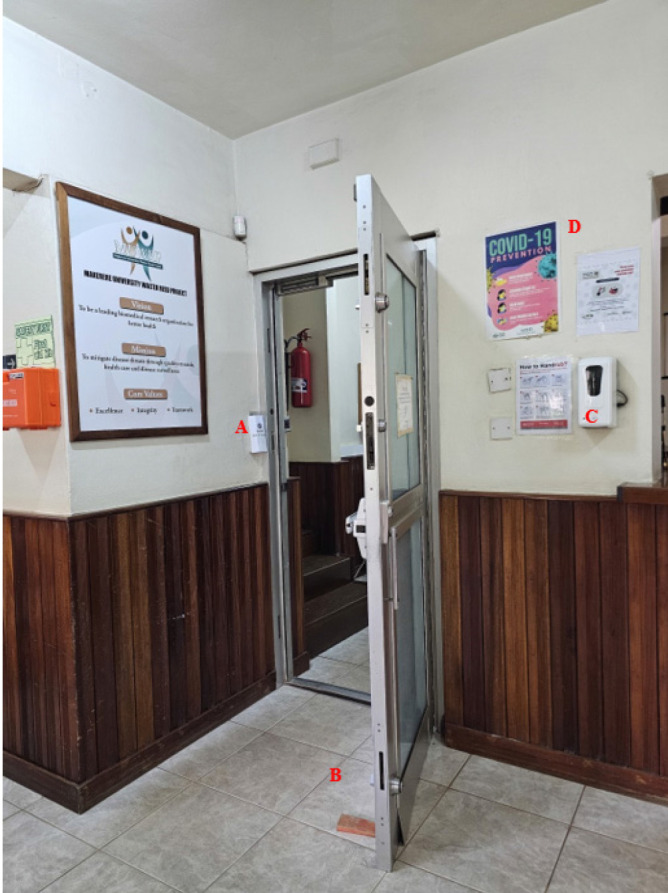
A Picture at MUWRP Showing: (A) Electronic Biometric Access System Sealed Off, (B) Access Door Kept Open, (C) Automatic Sanitiser, And (D) COVID-19 Prevention Information

Adjustments were undertaken to: (a) minimise the risk of staff interacting with high-risk contact points such as doorknobs and biometric access control devices (BACDs); Door stoppers were installed to improve air circulation and flow, and staff log in and access to premises through BACDs was halted, and all devices sealed off (Figure 1 (A)). (b) Minimise the risk of access to MUWRP’s premises by staff with COVID-19 like-symptoms; electronic temperature monitors were installed at entry points, and readings were recorded daily. (c) Lower interpersonal risk through installation of automatic alcohol sanitizers (Figure 1 (C)) at strategic points, procurement and provision of free personal use sanitizers, free face masks to all staff, prohibiting admission to premises of people not wearing masks, and providing free periodic sanitization of laptops by the information technology department.

#### Technological Modifications

This component focused on leveraging technology to: (a) minimise contact with contamination prone paper, and (b) reducing the odds of contact between the different staff. For this, (a) we transitioned to a fully digital operational model which effected the following innovations: (1) online purchase requests and order processing, (2) digitising job applications and talent management, (3) adopting secure, unlimited cloud data storage for seamless data access and management, (4) transforming the staff intranet into a comprehensive digital workspace, facilitating employee engagement and collaboration remotely, (5) and digitising time sheets and employee leave management. For (b) MUWRP introduced (1) virtual training, webinars, and meeting spaces, (2) a secure virtual private network (VPN) was established for the remote workforce, (3) a documentary style virtual tour of the research facility was created, enabling virtual clinical and lab audits from international standards accreditation bodies, and (4) 24 hour virtual support systems for remote workers were instituted.

#### Human Capital Adaptations

Under this component: (a) the number of staff allowed at their duty stations at any timepoint was cut to 30%, providing more room for social distancing from within the laboratories, clinics, and offices, (b) all staff with any COVID-19 like symptoms were expected to stay at home and report immediately to their IPC line lead for further investigations, and most importantly (c) all staff above sixty years, and anyone with chronic illnesses was approved to work from home. Technological enhancements mentioned above meant that there was smooth continuity.

#### Ensuring Continuity of all Arganizational Activities

The banning of public transport by the president of Uganda^24^ threatened the continuity of MUWRP’s activities since over 85% of all staff were dependent on public means to commute from home to their workstations. However, the transport department, and human resource office drafted a plan to map and schedule staff pickups. Staff from within the same vicinity were advised to walk to agreed pick-up points where they would be picked up and back by the organisation’s drivers. The organisation’s motor vehicles had government permits that allowed them to move during the lockdown.

### Timely Identification and Support Systems for Positive Staff

Under this pillar, the theory for action was threefold: (a) early identification of infected staff, (b) timely linkage to quality care and treatment, and (c) post treatment support systems and plans in case any staff passed on. The section below highlights the innovations, changes and actions that were undertaken.

#### Efforts for Early Identification

At the onset of the pandemic, early COVID-19 detection was seen as a key to survival, and MUWRP adopted this as one its priorities. Beyond temperature checks, management commissioned its MoH approved emerging infectious diseases laboratory to provide free testing services for any staff that experienced symptoms related to severe acute respiratory syndrome 2 (SARS-CoV-2). This was to take the burden of the costs of testing, which were then over $65 per test, off the staff.^28^

#### Timely Linkage to Quality Care and Treatment

Under this sub-pillar, the plan was to minimise the number of staff infected that progressed to severe COVID-19, and hence reduce to the minimum the risk of associated deaths. The following innovations were adopted: (1) the IPC focal person reached out to the ministry of health (MoH) to avail an ambulance to pick any MUWRP staff with COVID-19 to treatment centers, (2) some of MUWRP’s vehicles were earmarked as emergency vehicles for evacuation of positive staff, (3) discussions were held with health insurance providers to avail emergency ambulances for evacuation of staff for cases where organisational vehicles were unsuitable, (4) MUWRP and health insurance companies agreed to provide free healthcare to all MUWRP staff who fell victim, and (5) select hospitals were informed to book space for any staff that contracted COVID-19. This ensured that the referral pathway was smooth, and there were no delays in initiation of treatment for victims.

#### Support Systems for Recuperation and Casualty Management

For infected staff, plans were laid out to ensure complete recovery. (1) Daily follow up calls by the line IPC focal persons were made to assess progress, and for those exhibiting severe symptoms, plans were in place for evacuation to hospitals, (2) all infected staff were given 14 days off work for recovery, and (3) counselling services were availed by the organisation and health insurance providers for any persons that required psychosocial support.

### Sensitisation and Support for Prevention

This pillar focused on empowering all staff with knowledge, information, and skill to adopt risk minimisation behaviour, and positive attitudes towards vaccination, and making the vaccines accessible to staff and their family members.

#### Information, Education and Communication

**Figure 2:**
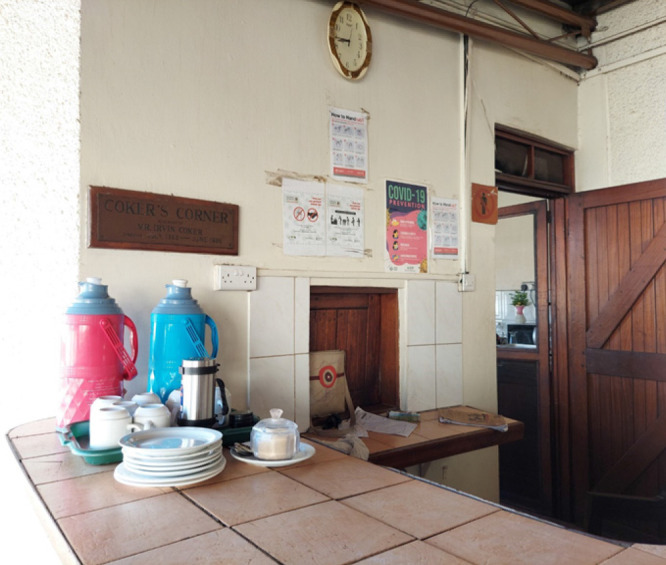
IPC Leaflets Hung at MUWRP’s Nakasero Canteen

**Figure 3:**
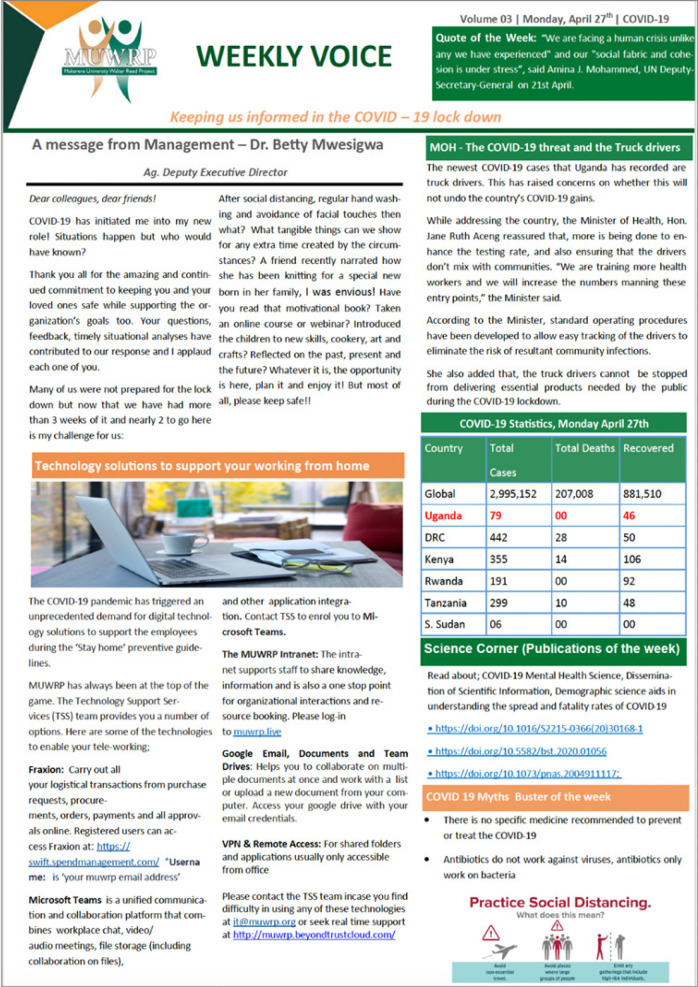
COVID-19 Newsletter; Volume 3 of April 27th, 2020

Innovations under this sub-pillar were: (a) COVID-19 related IPC materials were printed, laminated, and put at strategic points such as the dining area, reception, walkways, elevator steps, and in offices (Figure 2), (b) Weekly information sessions on national COVID-19 updates and how MUWRP was faring were held, and (c) the community department introduced a weekly newsletter to appraise staff at home on what was happening within the organisation and in the country. The newsletter was leveraged by staff to share their experiences of how the lockdown was treating them, and any other personal level innovations for making their life better while at home (Figure 3).

#### Vaccination for Prevention

Vaccines were another prevention strategy. The IPC committee came up with innovations anchored towards sensitisation about the vaccines, and making the vaccines available near to anyone that needed them. Hereunder: (1) four continuous medical education sessions focused on vaccine development, mechanism of action, reported adverse events following immunisation by each of the emergency use authorized vaccines, as well as the pros and cons of each were conducted, (2) management availed resources to ensure that free vaccines were secured from government and delivered at all MUWRP operation sites for ease access by staff, and (3) staff family members and relatives were allowed to access the same vaccines. This was very helpful during a time when vaccines were still hard to find as vaccinating family members was deemed as an indirect way of protecting staff.

### Leadership for Epidemic Management

**Figure 4:**
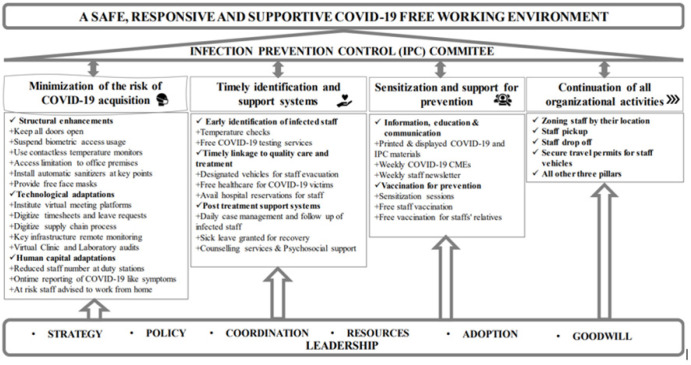
MUWRP’s Theory of Response During the COVID-19 Pandemic

Management in collaboration with the IPC committee led the strategic, operational, and co-ordination roles that supported execution of the four pillars. Hereunder: (a) The IPC committee developed and executed the MUWRP COVID-19 Risk Management Policy, (b) Weekly briefer meetings between management and the IPC committee were held to discuss progress on agreed upon activities, identify challenges, and come up with solutions. During these meetings, updates/briefs on sick staff were shared, and supportive actions were agreed upon, (c) Organisational resources such as laboratories, vehicles, funds, expertise, clinics, technology, communication lines, were leveraged to support any staff that caught COVID-19, and (d) engagements were held with the MoH to approve travel for essential MUWRP staff during the pandemic. For this, movement permit stickers were given to essential staff with vehicles to enable them commute when all other transport methods were halted (Figure 4).

### Data Analysis

Routine testing, positivity and treatment data collected from all staff was kept in a human resource tracking tool managed by the MUWRP human resource team. Deidentified data was abstracted, cleaned, and exported to STATA15.1 (Stata Corp, College Station, Texas, USA) for analysis, where descriptive and comparative analysis was conducted. The positivity rate was computed as a proportion of staff with an observed positive PCR test for SARS-CoV-2 across three segments corresponding to the national COVID-19 waves. The positivity rate across departments and cadres was also descriptively compared using proportions and graphical illustrations. The trend in SARS-CoV-2 across the three waves was computed using the chi-square statistic for the trend.

### Ethical Considerations

This paper describes MUWRP’s experience during the COVID-19 pandemic and highlights changes or innovations that were implemented through a phenomenological lens. The nasal swab sample collection from staff and data used in this paper was collected as part of a national public health response and thus did not merit institutional review board approval. That said, verbal consent was sought for all staff that were tested, and self-COVID-19 status reporting was voluntary. All staff data was strictly kept confidential per MUWRP data protection policies, and authors had only access to deidentified data.

## RESULTS

### SARS-CoV-2 Circulation among MUWRP Staff

Average SARS-CoV-2 positivity was 7% across the three waves, with most staff infected during wave one, and only 3.1% during wave three. Overall, males were more affected than females although there were more females affected during the second wave, though with no statistical significance in differences. Age-wise, staff within the 30 to 39 year category were the most affected group, followed by those within the 40 to 49, and 50 to 59 age categories (Table 2). There were no significant differences in positivity across all groups.

**Table 2:** SARS-CoV-2 Positivity at MUWRP

Factor	Staff n (%)	Positivity rate				*p-value* **
		Overall Positivity (%)*	Wave one, n (%)	Wave two, n (%)	Wave three, n (%)	
Overall	196 (100)	7.0	19 (9.7)	16 (8.2)	6 (3.1)	<0.010
Gender						
Male	106 (54.1)	7.6	13 (12.3)	7 (6.6)	4 (3.8)	0.019
Female	90 (45.9)	6.3	6 (6.7)	9 (10.0)	2 (2.2)	0.220
Age							
20–29	17 (8.7)	2.0	1 (5.9)	0 (0.0)	0 (0.0)	0.216
30–39	100 (51.0)	8.7	10 (10.0)	10 (10.0)	6 (6.0)	0.315
40–49	60 (30.6)	6.1	7 (11.7)	4 (6.7)	0 (0.0)	0.008
50–59	17 (8.7)	5.9	1 (5.9)	2 (11.8)	0 (0.0)	0.466
60–69	2 (1.0)	0.0	0 (0.0)	0 (0.0)	0 (0.0)	–

* Represents average positivity across the three waves

** p values were computed using Chi-square test for trend across the three waves

### COVID-19 and Job Status at MUWRP

**Figure 5:**
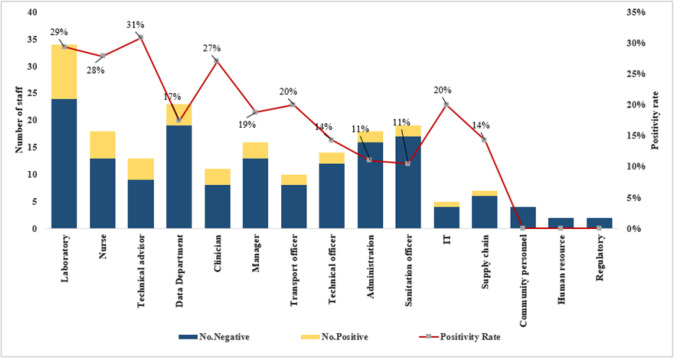
COVID-19 Positivity Rate by Cadre at MUWRP During the COVID-19 Pandemic

Technical advisors (field based staff working on the HIV/AIDS and surveillance projects) were the most affected group of staff with an overall 31% positivity followed by laboratory staff (29%), nurses (28%), clinicians (27%), transport officers (20%), information technology (IT) staff (20%), and managers (19%). The least affected categories were administrators, human resource specialists, the Kampala based community team, and the regulatory department (Figure 5).

### Severity of COVID-19 Cases at MUWRP

**Figure 6:**
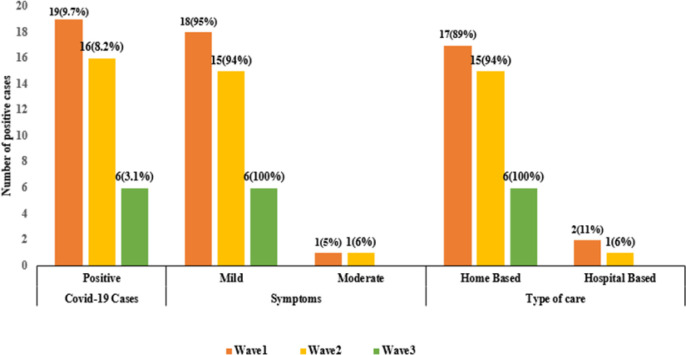
COVID-19 Positivity, Symptomatology, and Care at MUWRP

During wave one, positivity was at 9.7%, with 95% of all cases being mild, and two of the cases (11%) requiring admission to the hospital. During wave two, the positivity rate reduced to 8.2%, with 94% of cases being mild, and one case requiring hospitalization. In the third wave, only 3.1% of all staff were infected, and all cases were mild and managed at home (Figure 6). Across all waves, over 94.3% of all cases were managed through home-based care; whereby staff received their medication from their homes with no need for hospitalisation. Home-based care was associated with having a mild to moderate form of SARS-CoV-2.

### Uptake of Vaccination at MUWRP

**Figure 7:**
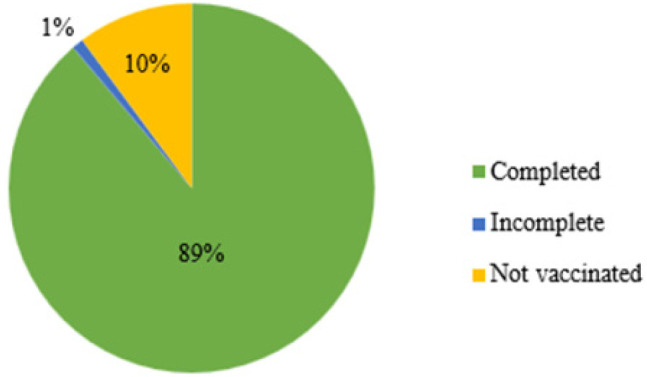
Vaccination Status of MUWRP Staff as of March 2023

Figure 7 shows the proportion of MUWRP staff that were vaccinated. By the time the WHO declared the end of COVID-19 as a global health emergency, 89% of all MUWRP staff were fully vaccinated, 1% were partially vaccinated, while 10% were not vaccinated.

## DISCUSSION

The emergence and source of COVID-19 continues to be a mystery, an indicator that we are likely to be faced by another outbreak. For this, any lessons, and best practices form a critical body of knowledge for all global public health players, more so NPHOs which continue to be relevant in the developing world’s context. In this section, we leverage existing literature to create a comparative discourse on the results of our risk minimisation efforts.

### COVID-19 at MUWRP, Uganda, and Globally

**Figure 8:**
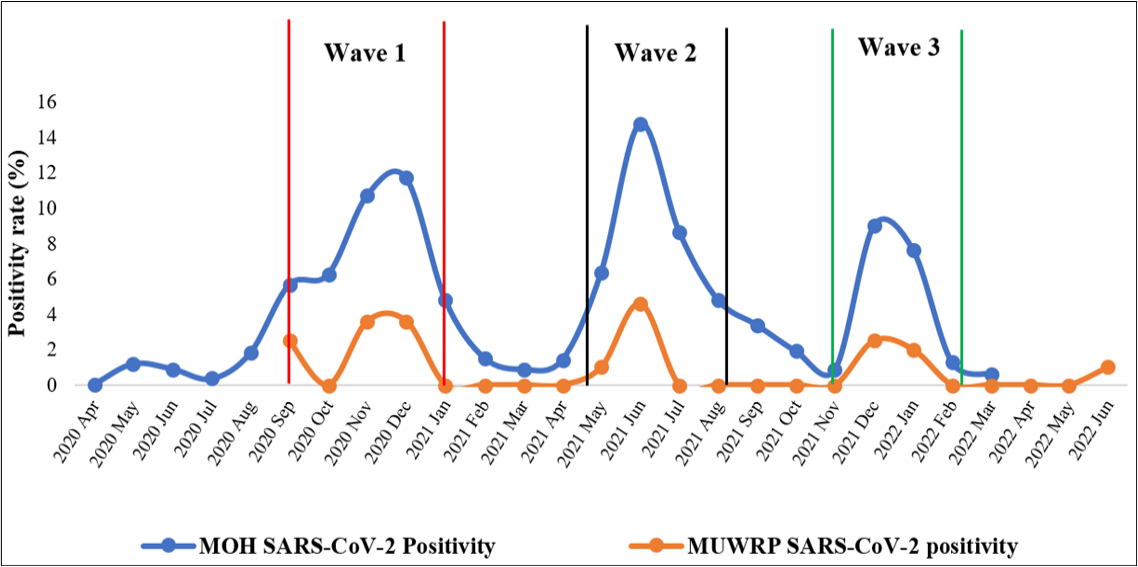
SARS-CoV-2 Circulation at MUWRP in Comparison to Uganda

MUWRP’s average positivity rate of 7% was low compared to the national average of 20%,^29^ and among contemporaries who performed comparable duties. This is further justified by Figure 8 which show that Ugandan ministry of health’s nationwide positivity rate was far higher. Further, seroprevalence among health care workers (HCWs) in south central Uganda was reported at 26.7%,^30^ and 22.6% in Brazil.^31^ This finding further strengthens the notion that our innovations played a major role in minimising the risk of SARS-CoV-2 among staff. The fact that males and staff within the 30 to 39 year age group were most affected is in line with other published studies in Uganda^23,32,33^ and globally.^34–36^ That said, circulation of COVID-19 was higher in the mentioned studies, further supporting the role of MUWRP’s interventions in minimising the risk.

MOH Data source @ www.health.go.ug/covid, accessed April 2023

### Employment and COVID-19

Frontline staff such as community-based HIV/AIDS technical advisors, nurses, clinicians, laboratory personnel and IT specialists had the highest positivity rates. This is because they were more in contact with patients, samples, and other health partners. For example, technical advisors followed up HIV/AIDS and TB patients in communities to refill their regimens, and manage any emergencies, while IT staff moved from desk to desk and sometimes to the field and staff homes to backstop challenges with the new adopted technology such as zoom. The high positivity among MUWRP frontline health workers mirrors a study conducted in Saudi Arabia that also found frontline health workers such as nurses and clinicians carrying a higher burden of COVID-19 compared to staff working in supportive departments such as logistics, human resource, maintenance and engineering,^37^ and other studies have reported even higher PCR positivity, and seropositivity among frontline health workers. That said, MUWRP’s positivity rate was lower compared to 26.7% seroprevalence among health workers in south central Uganda,^30^ the countrywide positivity rate of 20%,^29^ and 22.6% among health workers in Brazil,^31^ which further strengthens the institution of innovations as possible factors for the good performance.

### COVID-19 and Severity at MUWRP

Most COVID-19 cases (96.3%) at the organisation were mild, and 94.3% of all cases were managed through home-based care. Mild presentation was far higher at our organisation compared to 48.1% at one hospital in Kampala,^38^ and 68.1% at government health facilities.^33^ After controlling for age where MUWRP demographics do not differ significantly from national and African statistics,^39^ the high number of mild cases can be attributed to organisational vigilance through innovations such as intensified screening, early and accessible testing, and referral mechanisms. Moreover, early identification and treatment were known for better outcomes, and lesser odds of deterioration and spread.^40–43^ The many mild cases justify employing home based care for 94.33% of all cases. It is noteworthy that the three hospitalized staff were field frontline health workers, two of whom were above 47 years of age. Older age and increased exposure due to interaction with patients were known drivers for the infection.^44–47^ Our organisation did not report any SARS-CoV-2 related fatalities. This contrasts with the national rate of 2.2%, and 36.5% among admitted patients during the second wave.^39,48^ Whereas there are no documented studies on COVID-19 related loss of life among health care workers (HCWs) in Uganda, studies have reported mortality rates of up to 3.5%,^49^ 0.92% for a global systematic review,^50^ 0.33% in the United States of America (USA),^51^ and 0.3% for the USA, Italy and China.^52^ MUWRP’s initiatives such as daily temperature screening, free and accessible testing among others could have contributed to minimising the risks of death since they contributed to early identification of cases, and hence early treatment initiation, known protectors from severe ailment forms and death.^53,54^

### Vaccination at MUWRP

The 89% COVID-19 vaccination completion rate among MUWRP staff far contrasts the national rate which lingered between 19% and 35% by May 2023.^55–57^ Low uptake was also reported in neighboring countries such as Kenya (10% to 30%),^58,59^ Democratic Republic of Congo (less than 5% as of July 2022),^60^ and generally in the whole of Africa where vaccine hesitancy was widespread.^61,62^ The high uptake can thus be attributed to the innovations MUWRP introduced such as in depth discussions on the vaccines which clarified any misconceptions, and bringing the vaccines close to those in need, thus closing the access gap. Availability of different choices of vaccines could also have been a factor in increasing uptake given staff had an opportunity to choose which vaccine they preferred, moreover studies have shown Moderna and Pfizer to have a higher preference and uptake rate.^63^ All withstanding, our findings were in line with another study conducted in Uganda^64^ and other countries which showed relatively higher COVID-19 vaccine acceptance and completion rates among health care workers,^65^ although the MUWRP statistic still remained higher.

### Implications for NPHOs

The next pandemic is inevitable.^66–68^ It is only about “when” and “how” health players such as NPHOs will be organized to minimise their risks and impact and ensure continuity of their obligations in resource limited countries. Strong leadership and coordination have been discussed and reported to be critical for better COVID-19 response and overall organisational management and transformation.^69,70^ In this paper we have showcased the strategic and operational actions that any health implementing institution could leverage to ensure the safety of its employees operating during generalized outbreaks.

### Study Limitations

This paper showcases a combination of changes and innovations that were instituted at various pillar and sub-pillar levels with the overall goal of minimising the risks of acquisition of COVID-19 by MUWRP staff. Still, our improvement and capacity to minimise infection is attributable to the whole package of innovations we implemented. We did not delineate which changes were responsible for what improvement. However, the paper provides a foundation for future studies seeking to measure the extent of improvement per innovation MUWRP tested. Furthermore, data used in this submission was based on voluntary reporting by staff, leaving a possibility of underreporting. However, the IPC committee kept informing all staff of the importance of reporting when one is infected, and support such as long periods of leave to recover were seen as encouraging staff to inform management when they have COVID-19. Lastly, this study describes a phenomenological experience within a pandemic situation and hence would require systematic and planned studies to further validate our findings. Overall, our experience provides a benchmark and information on good practices and innovations that proved effective and can be adopted and adapted by NPHOs during the next pandemic.

## CONCLUSION

The COVID-19 pandemic caught the whole world unaware, and public health implementation challenges during this time affected NPHOs that had no prior experience in operating in such situations. Here, we have discussed the changes, innovations and actions instituted by MUWRP, one of the NPHOs in Uganda, and highlighted good leadership, cues for risk minimisation and continuity of health service delivery, early identification and support systems for positive staff, sensitization, and support for prevention as critical pillars for minimising the impact of the next outbreak on staff. We recommend more studies in this area, focusing on structured long-term assessment of how the different changes contributed to improvement, and how organisations are led towards these results.
